# Der Allgemeine Soziale Dienst in der Corona-Pandemie: Empirische Erkenntnisse zu den Arbeitsbedingungen und deren Auswirkungen auf die (professionelle) Soziale Arbeit im ASD

**DOI:** 10.1007/s12054-022-00485-0

**Published:** 2022-05-09

**Authors:** Nikolaus Meyer, Verena Klomann, Elke Alsago

**Affiliations:** 1 Fulda, Deutschland; 2 Darmstadt, Deutschland; 3 Berlin, Deutschland

**Keywords:** Allgemeiner Sozialer Dienst, ASD, Professionalität, Corona-Pandemie, Soziale Arbeit

## Abstract

Die Arbeitsbedingungen im Allgemeinen Sozialen Dienst (ASD) der Jugendämter und die Folgen für den Kinderschutz gerieten in der Corona-Pandemie schnell in den Fokus der öffentlichen Aufmerksamkeit und wurden kontrovers diskutiert. Der vorliegende Beitrag beleuchtet unter Bezugnahme statistischer Daten aus dem ersten und zweiten Lockdown die Arbeitsbedingungen und Kontaktmöglichkeiten in diesem Arbeitsfeld und differenziert hierbei die Einschätzung von Leitungs- und Fachkräften im ASD.

Die Arbeit im Allgemeinen Sozialen Dienst der Jugendämter wurde durch die Pandemie erheblich beeinflusst und hat sich bisweilen maßgeblich verändert. Leitungs- und Fachkräfte nehmen diese Entwicklungen unterschiedlich wahr.

Früh im Verlauf der Corona-Pandemie geriet eine zentrale Facette der Arbeit im Allgemeinen Sozialen Dienst (ASD)^1^ des Jugendamts – der Kinderschutz und die hiermit verbundene Garantenstellung – in den Fokus einer breiteren Öffentlichkeit (Zitelmann et al. 2020). Dabei schwankten die Positionierungen und Problemanzeigen zwischen einer wahrgenommenen unzureichenden Sicherstellung des Schutzauftrages (ebd.) und dem Bemühen um die Sicherung des „fachlichen Mindeststandard [s, A.d.A.] im Leistungsbereich […] und im Kinderschutz“ (BAG ASD 2020, S. 1).

Im Verlauf der Pandemie griffen empirische Untersuchungen die Situation im ASD auf. So untersuchte das Deutsche Jugendinstitut (DJI) die Fragen „der Aufrechterhaltung des Leistungsspektrums der Kinder- und Jugendhilfe, […] darunter auch zur Aufrechterhaltung des Kinderschutzes und zur Entwicklung von Fallzahlen“ (Mairhofer et al. 2020, S. 66). Zu diesem Zweck „wurden in einer bundesweiten Onlinebefragung bei allen 575 Jugendämter[n, A.d.A.] […] empirische Befunde zu Leistungen und zur Arbeitsweise der Kinder- und Jugendhilfe in dieser Krisensituation erhoben“ (ebd., S. 66). An der Befragung nahmen 371 oder 65 % aller Jugendämter teil (ebd.). Die Erhebung bezog sich auf die Leitungsperson des jeweiligen Jugendamtes. Hinsichtlich der eingelangten Hinweise auf mögliche Kindeswohlgefährdungen gaben 55 % der befragten Jugendamtsleitungen an, dass diese Zahl im Vergleich zur Zeit vor der Corona-Krise konstant geblieben ist, bei 25 % ist sie zurückgegangen (ebd., S. 5). Ein Trend, der sich auf Jahressicht nicht bestätigte: Die Anzahl der Gefährdungseinschätzungen stieg im Jahr 2020 um 9 % an (Destatis 2021). Bezüglich einer fachlich angezeigten Inaugenscheinnahme im Rahmen des Gefährdungseinschätzungsprozesses gem. §8a Abs. 1 SGB VIII verwiesen 98 % der Jugendämter im Befragungszeitraum April/Mai 2020 darauf, weiterhin Hausbesuche durchzuführen oder Termine im Jugendamt zu realisieren (49 %). Überdies nutzen 13 % der Jugendämter digitale Medien und 1 % führte an, dass Inaugenscheinnahmen vorübergehend ausgesetzt wurden (ebd., S. 37). Weiter gab ein Drittel der Jugendämter an, dass sie in der weiteren Arbeit aufgrund der Beschränkungen auf direkten, persönlichen Kontakt zu ihren Adressat_innen verzichtet zu haben, wodurch rund 47 % der Jugendämter anderweitige Kommunikationsmodi, wie Online‑, Telefon- und Chatberatung, genutzt haben (ebd.).

Eine weitere Studie des DJI (2020) untersuchte, mithilfe von „leitfadengestützten Telefoninterviews mit den ASD-Gruppenleitungen aus Jugendämtern“ (ebd.)^2^, die „Auswirkungen […] [der, A.d.A.] COVID-19-Pandemie auf die Arbeit im Kinderschutz“ (Gerber und Jentsch 2021, S. 294). Im Kern wurden die vorhandenen Schutzkonzepte im Pandemieverlauf in den Jugendämtern teilweise beibehalten und gleichzeitig modifiziert (ebd.). Dies wurde vor allem aufgrund des Wegfalls bisheriger Unterstützungsstrukturen, wie der KiTa, oder der Verzögerungen bei der Einrichtung der Notbetreuung nötig und erforderte nicht selten alternative Überbrückungsoptionen, die häufig durch Fachkräfte des ASDs realisiert wurden (ebd., S. 295). Gleichzeitig verweist die Studie auf „atmende Verfahren“ (Meysen und Schönecker 2021, S. 494), also auf Veränderungen in den internen Prozessen nach §8a SGB VIII: „Im ASD wurde einerseits [sic!] an den geltenden Standards […] festgehalten und gleichzeitig im Lichte des notwendigen Infektionsschutzes Anpassungen vorgenommen. Das hat zu stärker einzelfallorientierten Abwägungsprozessen und Vorgehensweisen sowie einer Verschiebung von Prioritäten geführt“ (ebd., S. 494).

Eine weitere Studie interviewte mithilfe einer „Online-Befragung […] 1744 Mitarbeitende aus über 300 der 559 Jugendämter in Deutschland“ (ISM 2021, S. 30).^3^ An der Erhebung nahmen sowohl Fach- als auch Leitungskräfte aus dem ASD teil – eine Differenzierung der Befunde nach diesen beiden Gruppen erfolgte im publizierten Abschlussbericht jedoch nicht. Hinsichtlich der Kontaktarten und -häufigkeiten gaben 80 bis 90 % der befragten ASD-Mitarbeiter_innen an, dass „sich die Erreichbarkeit von Jugendlichen zwischen 14 bis unter 18 Jahren, von Familien in prekären Lebenslagen und von psychisch erkrankten Eltern verschlechtert“ habe (ebd., S. 31). Weiter wird hervorgehoben, dass die Jugendämter „durch die Corona-Pandemie über alle Lebensbereiche hinweg negative Veränderungen im Leben von Kindern, Jugendlichen und jungen Erwachsenen“ und in der Folge „einen (starken) Mehrbedarf in allen aufgeführten Leistungsbereichen der Kinder- und Jugendhilfe“ (ebd., S. 20) sehen. Daneben verweisen die Untersuchungsergebnisse auf Veränderungsbedarfe hinsichtlich der Kooperationsbeziehungen und -strukturen (ebd. S. 22 f.).

Die benannten Untersuchungen verdeutlichen zentrale Aspekte der Situation in den ASDn der Jugendämter während der Corona-Pandemie. Hierbei greifen sie jedoch entweder die Sicht der Leitungsebene, jene der Fachkräfte auf, oder differenzieren bezogen auf beide Beschäftigtengruppen die Ergebnisse nicht. Am Beispiel des Frankfurter Jugendamts – hier berichtet die Behördenleiterin über andere Erfahrungen im Pandemie-Alltag (Lohse 2020) als eine Fachkraft (verdi 2020) – zeigt sich, dass die Perspektiven sehr unterschiedlich sein können. Es bedarf also der Erfassung und Würdigung beider Perspektiven, um die Veränderungen im Alltag des ASD adäquat beschreiben und ihre künftige Relevanz für den Prozess der Professionalisierung deuten zu können.

## Ausgewählte empirische Erkenntnisse

Der vorliegende Beitrag geht der Frage nach, wie Leitungs- und Fachkräfte aus dem ASD die pandemie-indizierten Entwicklungen der Arbeitsbedingungen sowie der Kontakt- und Interaktionsmöglichkeiten einschätzen. Grundlage hierfür sind zwei quantitative Online-Erhebungen^4^ während des ersten und zweiten Lockdowns.^5^ Auf Basis einer Sonderauswertung dieser nicht-repräsentativen Daten werden die Wahrnehmungen der Leitungskräfte (*n* = 20 in der Befragung während des ersten sowie *n* = 32 während des zweiten Lockdowns) sowie jene der Fachkräfte (*n* = 89 in der Befragung während des ersten sowie *n* = 218 während des zweiten Lockdowns) im Allgemeinen Sozialen Dienst der Jugendämter differenziert beleuchtet.^6^ Der Umfang des Datensatzes, dessen sind sich die Autor_innen bewusst, ist aus Sicht der quantitativen empirischen Sozialforschung nicht ausreichend, um verallgemeinerbare Tendenzen abzuleiten. Allerdings zeigen die vorliegenden statistischen Befragungsdaten ähnliche Einschätzungen wie die eingangs zitierten Studien und sind als Ergänzung dieser zu betrachten. Außerdem verweist dieser Beitrag auf ein bestehendes Dilemma: Die unzureichende Differenzierung der Perspektiven verschiedener Statusgruppen im ASD verzerrt die aktuell vorhandene Beschreibung der Lage im ASD während der Corona-Pandemie. Es bleibt ein dringend zu klärendes Desiderat.

## Systemrelevanz und Öffnungsquote im ASD

**Figure 1:**
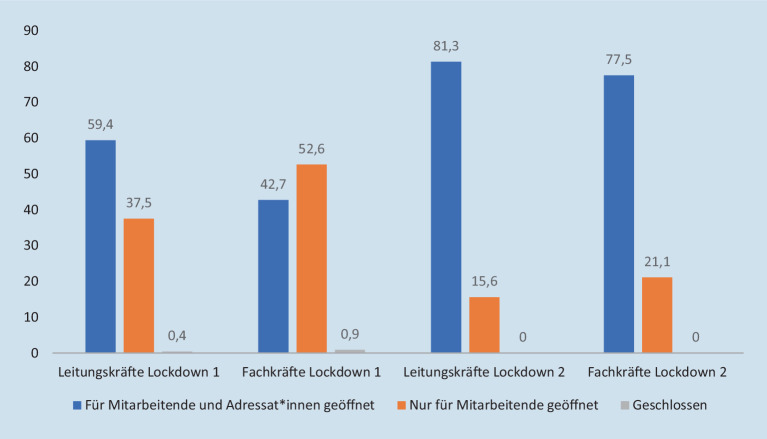

In der ersten Phase der Corona-Pandemie dominierte in der Sozialen Arbeit, wie gesamtgesellschaftlich, zunächst die Unsicherheit im Umgang mit den Schutzmaßnahmen sowie die Frage, welche Maßnahmen angemessen sind. Im ersten Lockdown gaben 59,4 % der Leitungskräfte an, dass die Räumlichkeiten für Adressat_innen, wie Mitarbeitende, geöffnet waren, während diese Antwort nur 42,7 % der Fachkräfte wählten (Abb. 1).^7^ Gleichzeitig wiesen die Befragten – zumeist mit Blick auf Desinfektionsmittel, Mund-Nasen-Schutz etc. – auf erhebliche Mängel bei Schutzmaterialien hin. 26,8 % der Fachkräfte und 15,8 % der Leitungskräfte gaben an, keinen 1,5 m-Abstand zu Adressat_innen einhalten zu können. Hierdurch lassen sich möglicherweise die geringeren Öffnungsquoten im ersten Lockdown im Vergleich zum zweiten erklären (s. Abb. 1).

Hinsichtlich der Öffnungsquote gaben im zweiten Lockdown mehr als drei Viertel aller Leitungs- (81,3 %) sowie Fachkräfte (77,5 %) an, dass die Räumlichkeiten des jeweiligen ASDs sowohl für Mitarbeitende als auch für Adressat_innen geöffnet sind. Gleichzeitig wiesen Leitungs- (87,5 %) und Fachkräfte (74,3 %) mehrheitlich darauf hin, dass die dafür notwendigen Schutzkonzepte aus der ersten und eher plötzlichen Schließungsphase der Pandemie stammen und zumeist unverändert im zweiten Lockdown Anwendung gefunden haben. Diese Schutzmaßnahmen werden überwiegend negativ bewertet. 78,9 % der Fach- und 75 % der Leitungskräfte sehen sich durch diese an der Ausübung ihrer Tätigkeit gehindert.

## Arbeitsbeziehungen und -weisen

Während des ersten Lockdowns lassen sich drei Dimensionen der Veränderungen der Arbeitsbedingungen beschreiben, die unmittelbare Auswirkungen auf die Arbeitsbeziehungen zwischen Leitungs‑/Fachkräften einerseits sowie Adressat_innen andererseits haben. So nehmen die Befragten in dieser ersten Phase der Corona-Pandemie zunächst eine Art Stagnation bei der Nachfrage nach ihren Angeboten (Leitungskräfte: 84,2 %; Fachkräfte: 75,7 %) sowie parallel eine Abnahme des Kontakts zu den Adressat_innen während der zum Befragungszeitpunkt zurückliegenden sieben Tage wahr (Leitungskräfte: 30 %; Fachkräfte: 27,6 %). Eine weitere Änderung im professionellen Handeln ergibt sich aus dem Umstand, dass 26,3 % der Leitungskräfte sowie 15 % der Fachkräfte darauf verweisen, Hilfen früher als üblich zu beenden. Diese Entwicklung änderte sich im zweiten Lockdown vollständig: 35,5 % der Leitungs- und 46,1 % der Fachkräfte sehen eine wachsende Nachfrage nach Angeboten des ASDs und parallel einen Anstieg der Kontakthäufigkeit zu den Adressat_innen. Insofern sind im zweiten Lockdown 29,4 % der Fachkräfte mit mehr Adressat_innenkontakten als vor der Pandemie konfrontiert, wohingegen dies nur 9,4 % der Leitungskräfte wahrnehmen.

**Figure 2:**
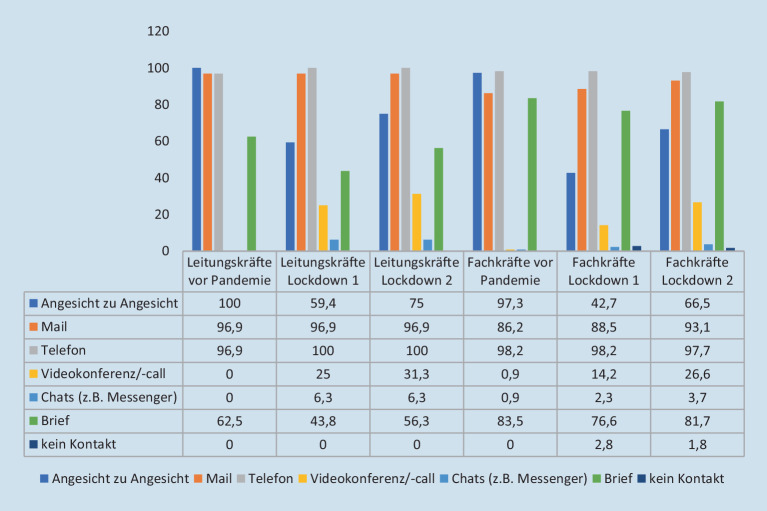

Hinsichtlich des Kommunikationsmodus sowie etwaiger Veränderungen während der Pandemie variieren die Einschätzungen der Leitungs- und der Fachkräfte (s. Abb. 2).

Leitungs- (75 %) und Fachkräfte (77,9 %) verweisen im zweiten Lockdown insbesondere auf grundlegende Veränderungen in der Kommunikation mit den bereits vor der Pandemie bekannten Adressat_innen und geben an, dass sich deren Problemlagen verstärkt hätten (Leitungskräfte: 70 %; Fachkräfte: 76,4 %) und das Armutsrisiko insgesamt gestiegen sei (Leitungskräfte: 76,9 %; Fachkräfte: 77,5 %). Auf die Exklusionsmechanismen von Armut und deren Auswirkungen in der konkreten Tätigkeit mit den Adressat_innen haben die Mitarbeitenden des ASDs in den offenen Antworten bereits im ersten Lockdown hingewiesen: Viele Adressat_innen verfügten nicht über die technisch erforderliche Infrastruktur. Zudem könne man weder am Telefon noch mit digitalen Tools die notwendige Vertrauensbasis für ein Arbeitsbündnis schaffen. Auf diese Weise entstünde von den beteiligten Menschen kein „Bild“. Digitale Medien schaffen neue Sprachbarrieren. Der Wegfall der Mimik der Adressat_innen mache Einschätzungen schwieriger. All das führe dazu, dass weniger dringliche Fälle in der Pandemie kaum bearbeitet werden und sich so zu Gefährdungslagen für Kinder und Jugendliche entwickeln könnten. Da die Fachkraft aus Gründen des Gesundheitsschutzes Termine allein wahrnehmen oder/zudem die Notwendigkeit des Besuchs schriftlich begründen müsse, sei hier eine entsprechende Abwägung notwendig. Die Verantwortung hierfür obliege der Fachkraft, wodurch das Gefühl des Alleingelassen-Werdens mit der Entscheidung und der Balance zwischen Kinder‑, Eigen- sowie Angehörigen- und Kolleg_innenschutz entstehe.

In den offenen Antworten im zweiten Lockdown weisen die Fachkräfte auf die problematischen Folgen dieser Entwicklung hin: So führten die Schutzmaßnahmen zu erschwerten Kommunikationsbedingungen, welche die ohnehin durch die Pandemie und den möglichen gesundheitlichen Folgen ‚verängstigten‘ Adressat_innen weiter aus der Arbeitsbeziehung drängten. Aus diesen Gründen sowie den nicht vorhandenen technischen Ressourcen nehme der Kontakt zu den Adressat_innen ab, was sich durch fehlende Hausbesuche sowie den sich reduzierenden Kontakt ‚Angesicht zu Angesicht‘ weiter potenziere.

## Digitalisierung

Die Nutzung digitaler Medien erlebt aus Sicht der Leitungskräfte seit Ausbruch der Corona-Pandemie ein moderates Wachstum (s. Abb. 2). Zudem konstatieren sie kaum Änderungen zwischen erstem und zweitem Lockdown. Die Leitungskräfte benennen im ersten Lockdown eine erhebliche Abnahme der Bedeutung von Briefen (−18,7 %), die im zweiten Lockdown abgeschwächt anhielt (−6,2 %). Weiter wird eine zunehmende Bedeutung von Videokonferenzen, wie Chats, von den Leitungskräften attestiert, wobei ein Wachstum bei Videokonferenzen zwischen den Lockdown-Phasen benannt wird. Der Anteil der Hauptform sozialarbeiterischen Handelns, der Kontakt von Angesicht zu Angesicht, sinkt im ersten Lockdown und steigt trotz der hohen Öffnungsquote aus Sicht der Leitungskräfte nicht wieder auf das vorpandemische Niveau an.

Eine ähnliche Einschätzung der Entwicklung des Modus der Kontaktaufnahme liegt bei den Beschäftigten vor: Die Interaktion von Angesicht zu Angesicht sowie Telefonate und Briefe als Kommunikationsmittel seien nach dem Einbruch im ersten Lockdown nicht wieder angestiegen. Demgegenüber verweisen die Beschäftigten auf eine Zunahme der digitalen Kontaktmodi im Verlauf der Pandemie.

Insgesamt rechnet jede zweite befragte Person (Leitungskräfte: 55,2 %; Fachkräfte: 51,9 %) mit einer weiterwachsenden Notwendigkeit zur digitalen Kommunikation mit den Adressat_innen. Fast drei Viertel der ASD-Mitarbeitenden gibt an, über das Wissen zur Gestaltung (Leitungskräfte: 68,8 %; Fachkräfte: 71,1 %) ebendieser zu verfügen – allerdings haben nur 19,3 % der Fachkräfte die nötige Technologie von ihrem Arbeit‑/Dienstgeber erhalten, um digitale Tools einzusetzen (Leitungskräfte: 15,6 %). Die jeweiligen Vorgesetzten können bzgl. der Digitalisierung als ein relevantes Nadelöhr bezeichnet werden: Während sich ‚nur‘ 44,4 % der Fachkräfte von ihren jeweiligen Vorgesetzen beim Einsatz von digitalen Technologien unterstützt fühlen, ist dies bei 68,8 % der Leitungskräfte der Fall.

## Austausch mit den Kolleg_innen und Kooperationspartner_innen

Eine wesentliche Veränderung ist auch bzgl. der fachlichen Kommunikation mit den Kolleg_innen zu verzeichnen: Mehr als drei Viertel aller Befragten nehmen einen veränderten fachlichen Austausch mit den Kolleg_innen (Leitungskräfte: 90,6 %; Fachkräfte: 84,8 %) ebenso wie mit den Vorgesetzten wahr (Leitungskräfte: 65,6 %; Fachkräfte: 63,1 %), was durch die starke Veränderung der Zusammenarbeit mit anderen Institutionen zusätzlich verschärft wird (Leitungskräfte: 71,9 %; Fachkräfte: 76,3 %). So wird in den offenen Antworten bemängelt, dass der Wegfall der personalen Interaktion schnelle und unbürokratische Vorgehensweisen zum Nutzen der Adressat_innen unmöglich mache. Überdies weisen einige der befragten Fachkräfte darauf hin, dass durch die digitale Kommunikation kein „Eindruck“ mehr von den Fachkräften anderer Institutionen in komplexen Fällen entstünde, obwohl dieser im Hilfeprozess nicht unerheblich sei. In der eigenen Einrichtung entfielen Supervisionen, Teamsitzungen oder Fortbildungen, sodass wichtige Reflexionsprozesse weder innerhalb des Teams noch mit den Kooperationspartner_innen stattfanden.

## Abläufe und Standards

Parallel zu den vorangegangenen Änderungen in den Arbeitsweisen und -beziehungen sind bei den Abläufen und Standardvorgehensweisen Modifikationen zu verzeichnen, wobei keine Bedingungszusammenhänge hergeleitet werden können. 100 % der Leitungs- sowie 90,4 % der Fachkräfte weisen darauf im zweiten Lockdown hin und beschreiben diese Änderungen in den offenen Antworten anschaulich:

- Die *Leitungskräfte* beklagen eine deutliche Zunahme der Arbeitszeit, was sich in den quantitativen Ergebnissen darstellen lässt (34,4 %). Als Erklärung werden vor allem die fehlende digitale Ausstattung sowie die Möglichkeit zum Austausch mit den Kolleg_innen bei gleichzeitig steigender „Bedürftigkeit“ und die erschwerte Kommunikation angegeben. Insgesamt bemängeln die Leitungskräfte die Änderungen des professionellen Handelns bedingt durch rechtliche Pandemie-Verordnungen und betonen, dass etablierte Standards problematische Abwandlungen erfahren.
- Die *Fachkräfte* verweisen ebenfalls auf negative Modifikationen der Standardabläufe und -verfahren. Sie sprechen über eine erhebliche Raumnot in ihren Gebäuden. Das ohnehin eingeschränkte Raumangebot erweise sich unter den Maßgaben von Mindestabständen und Kontaktbeschränkungen als problematisch. Parallel wurden in zahlreichen Einrichtungen offene Sprechstunden ersatzlos gestrichen, anstatt sie durch andere Angebote zu ersetzen. Eine Vielzahl von Entscheidungen in Hilfeprozessen werde im zweiten Lockdown nach Aktenlage getroffen, ohne mit den Adressat_innen oder anderen sozialpädagogischen Fachkräften gesprochen zu haben. Diese Steuerung der Fälle ohne persönlichen Kontakt problematisieren die Fachkräfte. Sie weisen auch darauf hin, dass nur Krisengespräche, Maßnahmen zum Kinderschutz und Gerichtstermine bearbeitet werden und alle anderen Aspekte unbearbeitet bleiben. Erschwerend käme hinzu, dass der ASD insgesamt schlechter als vor der Pandemie erreichbar sei. Zudem gäbe es Verzögerungen in der Bearbeitung von Fällen und einige ASDs seien nach außen „abgeschottet“.

Auf eine Veränderung von Standards und Arbeitsabläufen verweisen ebenso die quantitativen Daten aus der zweiten Lockdown-Phase: Hier geben 80 % der Befragten an, während der Pandemie neue Aufgaben übernommen zu haben.

## Arbeit im Homeoffice

**Figure 3:**
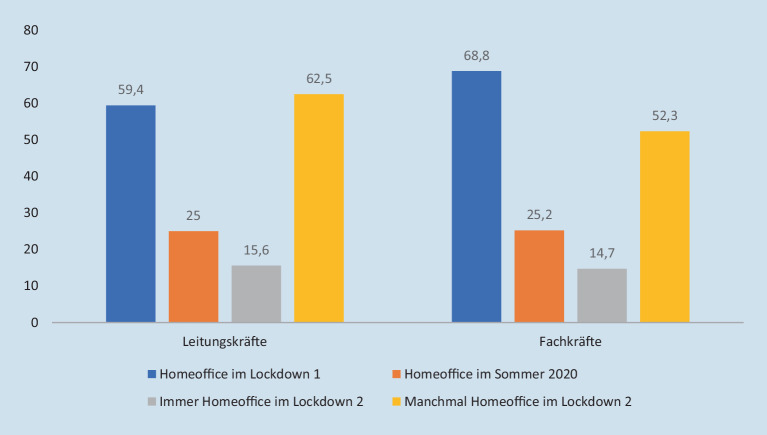

Ein wesentlicher Grund zur Notwendigkeit von veränderten Abläufen ist, neben den Schutzmaßnahmen und ihren Auswirkungen, die Arbeit im Homeoffice: Für eine Mehrheit aller Mitarbeitenden bestand zum Befragungszeitpunkt im ersten Lockdown die Möglichkeit zum Homeoffice (s. Abb. 3), wobei die übergroße Mehrheit der Befragten (Leitungskräfte: 100 %; Fachkräfte: 84,9 %) angibt, dass zu Beginn der Pandemie keine technischen Möglichkeiten zur Arbeit im Homeoffice bestanden hätten.

Während im ersten Lockdown vor allem Fachkräfte im Homeoffice tätig waren, hat sich dieser Anteil im Pandemieverlauf verändert. Gerade der Anteil von Leitungs- und Fachkräften, die manchmal, beispielsweise im Rahmen von sogenannten Kohorten-Regelungen zur Vermeidung von krankheitsbedingten Totalausfällen, im Homeoffice tätig sind, ist im zweiten Lockdown gestiegen.

Im zweiten Lockdown sinkt die Zahl der Beschäftigten, die dauerhaft im Homeoffice arbeiten. Diese Verteilung entspricht in etwa dem Verhältnis aller im öffentlichen Dienst beschäftigten Befragten (Meyer und Alsago 2021c). Die Werte weichen insgesamt allerdings von den Befragungsergebnissen zum Homeoffice in der Sozialen Arbeit ab (Meyer und Alsago 2021a), welche niedrigere Homeoffice-Raten aufweist. Sofern ins Gedächtnis gerückt wird, dass die Bedarfe der Adressat_innen während des zweiten Lockdowns von den befragten Fachkräften als gestiegen eingeordnet werden, diese aber weniger direkt erreichbar sind und weniger für eine entsprechende Interaktion zur Verfügung stehen, dann verweist dies auf eine erhebliche Diskrepanz in der Passung von Nachfrage und Angebot. Dies führt bspw. dazu, dass bereits eingeleitete Hilfen ohne erneuten persönlichen Kontakt weitergeführt werden.

## Arbeitszufriedenheit

Insgesamt ist die Stimmung unter den Leitungskräften bezogen auf die Arbeitsbedingungen wesentlich besser als bei den Fachkräften. Obwohl 37,5 % der Leitungs- und 58,1 % der Fachkräfte – also mehr als jede zweite befragte Person im ASD – angeben, dass sich die Arbeitsbedingungen während der Corona-Pandemie verschlechtert haben und sich nahezu jede zweite Person mindestens belastet fühlt (Leitungskräfte: 53,1 %; Fachkräfte: 62,8 %),^8^ denken nur 6,3 % (Leitungskräfte) bzw. 27,5 % über einen Stellenwechsel nach. ‚Nur‘ ist dabei mit Blick auf ein Viertel der befragten Personen auf der Fachkraft-Ebene (27,5 %) euphemistisch ausgedrückt und betont vielmehr die hohe Belastung. Gleichwohl ist der Wert im ASD niedriger als in der Kinder- und Jugendhilfe (ebd.) oder der Sozialen Arbeit insgesamt (Meyer und Alsago 2021a).

In den offenen Antworten im zweiten Lockdown wird diese ‚gefühlte‘ Entkräftung vor allem mit der hohen Zahl an zu vertretenden Kolleg_innen durch die Fachkräfte sowie den allgemeinen Personalmangel begründet. Darüber hinaus fordert der erhöhte Aufwand in der Organisation alltäglicher Tätigkeiten (z. B. adäquat große Räume zu finden, Flächen zu desinfizieren, Einarbeitung in neue coronabedingte Formulare zur Dokumentation von Einschätzungen im Kontext von §8a SGB VIII oder auch Dienstreiseanträgen) in der Wahrnehmung der Fachkräfte seinen Tribut. Nicht zuletzt erschöpfe die Fachkräfte primär die Gesprächssituation mit den Adressat_innen: Sie tragen zwar die Fallverantwortung, können durch institutionelle Bedingungen, wie verordnete Schutzmaßnahmen, jedoch nicht nach den fachlichen Standards (z. B. mit Blick auf Gesprächsführungstechniken) agieren. Parallel schaffen die neuen Arbeitsbedingungen auch Veränderungsprozesse in den Teams, was in den offenen Antworten als „Auseinanderbrechen von Teamstrukturen“ beschrieben wird.

## Blick in die Zukunft

Die Mitarbeitenden rechnen insgesamt mit einem Anwachsen der Aufgaben im ASD nach Ende der Pandemie (Leitungskräfte: 56,3 %; Fachkräfte: 60,1 %). Dies könnte weitere Beanspruchungen für ohnehin bereits belastete Mitarbeitende in der Zukunft beinhalten und die Zunahme einer Entkräftung sowie die Abnahme von Arbeitszufriedenheit begünstigen. Inwieweit es möglich sein wird, auf die von den Mitarbeitenden wahrgenommene Verschlechterung der Lebensbedingungen der Adressat_innen einzugehen, bleibt in Anbetracht der knappen personellen sowie technischen Ressourcen abzuwarten. All dies betont die Notwendigkeit einer entsprechenden Auseinandersetzung mit den Stellenprofilen, Zuständigkeitsregelungen und der Personalbemessung.

Die vorliegenden empirischen Daten verweisen zudem darauf, dass sowohl die Zusammenarbeit innerhalb des ASD als auch darüber hinaus, durch gravierende Veränderungen geprägt war und vermutlich durch erhebliche Verwerfungen begleitet wurde. So beschreiben einige Leitungskräfte beispielsweise, dass sie ihre Vorgesetzten während der Organisation technischer Ausstattung nicht mehr erreichen konnten oder sie in kurzer Folge mit unterschiedlichen Anweisungen konfrontiert und zur sofortigen Umsetzung aufgefordert wurden. Auch die Fachkräfte beziehen sich in den offenen Antworten im ersten Lockdown auf die teils chaotischen Zustände. Gleichzeitig lassen sich diese Gegebenheiten, dies zeigt der vorliegende Beitrag anhand der Differenzierung nach Statusgruppen, mit den vorhandenen empirischen Studien, die zumeist keinen Unterschied machen oder lediglich Führungskräfte befragen, derzeit weder genau beschreiben noch in den Dimensionen verallgemeinern.

Neben diesen Befunden stellt sich die Frage, welche weiteren Erkenntnisse die Auseinandersetzung mit den hier vorliegenden quantitativen Befragungsdaten – trotz der geringen Stichprobengröße – bringt. Ein forschungsmethodisches Phänomen ist für die gesamte Wahrnehmung der Arbeit im ASD während der Corona-Pandemie in diesem Beitrag als entscheidend herausgearbeitet worden: Tendenziell betrachten Leitungskräfte im ASD die Lage weniger negativ als Fachkräfte.^9^ Damit einhergehend drängen sich einerseits Fragen nach der Begründung für die unterschiedliche Einschätzung, andererseits auch nach der Aussagekraft vorhandener Forschungsergebnisse, die auf unterschiedliche Weise (quantitativ und qualitativ) Daten bei/mit den Leitungskräften aus den Jugendämtern erhoben haben, auf. Diese geben die Perspektive der Leitungskräfte wieder, lassen aber nur sehr begrenzt Rückschlüsse zur Situation der Beschäftigten im ASD sowie zur tatsächlichen Aufgabenwahrnehmungen auf Sachbearbeitungsebene wie bspw. im Kinderschutz etc. zu. Für den letztgenannten Phänomenbereich wäre gerade die Zusammenführung zwischen diesen verschiedenen hierarchischen Ebenen bedeutungsvoll. Darüber hinaus bleiben Fragen der Eingebundenheit von Leitungskräften in das Erleben des konkreten Alltags mit den Adressat_innen durch die Fachkräfte bis dato unberücksichtigt. So können die bisherigen Erkenntnisse kaum den tatsächlichen Alltag an der ASD-Basis in Deutschland während der Pandemie abbilden. Diese Situation kann im vorliegenden Beitrag jedoch nicht komplett ausgeleuchtet werden. So fehlt beispielsweise die Perspektive der Adressat_innen.

Ebenso erlauben die vorliegenden Daten keine Einschätzung zur Realisierung des Kinderschutzes während der beiden Lockdownphasen. Erkennbar sind jedoch die ambivalenten Veränderungen im professionellen Handeln von Leitungs- und auch Fachkräften Obgleich hier nur Einschätzungen aus einem Bruchteil der Jugendämter vorliegen, zeigen diese, dass die Devise nach der Pandemie weder „Augen zu und durch!“ noch im Anschluss „Weiter so!“ lauten kann. Vielmehr wird es darum gehen müssen, die Erfahrungen und Entwicklungen aus der Pandemie aktiv für die professionelle Weiterentwicklung der Sozialen Arbeit in den Allgemeinen Sozialen Diensten der Jugendämter zu nutzen. Die skizzierten Befunde und die sich darin befindlichen Impulse können und wollen hierzu einen Beitrag leisten.

## Footnotes

1 Der Allgemeine Soziale Dienst der Jugendämter (ASD) – auch Kommunaler, Regionaler oder Bezirkssozialdienst genannt – ist der zentrale Basisdienst im Rahmen der Kinder- und Jugendhilfe: Hier sind sowohl Unterstützung, Entlastung und Beratung der Adressat*innen (AGJ 2010) als auch die Wahrnehmung hoheitlicher Aufgaben, also der Schutz von Kindern und Jugendlichen, angesiedelt. Ein allgemeingültiges Standardprofil für den ASD existiert nicht (vgl. vertiefend Klomann 2014).

2 Diese „wurden zu zwei Zeitpunkten (Juli bis September 2020 und Oktober bis Dezember 2020)“ (Meysen und Schönecker 2021, S. 494) geführt.

3 Hinweis: In den zitierten Studien werden unterschiedliche Zahl der in der BRD vorhandenen Jugendämter angenommen.

4 Nach der ersten Erhebung zwischen dem 7. und 15. April 2020 lagen 1867 verwertbare Fragebögen vor (Buschle und Meyer 2020; Meyer und Buschle 2021). An der zweiten Befragung nahmen zwischen dem 9. November und 6. Dezember 2020 3064 Beschäftigte in der Sozialen Arbeit teil (Meyer und Alsago 2021a). Die fehlende Repräsentativität begründet sich in der weiterhin fehlenden Dauerbeobachtung der *gesamten* Sozialen Arbeit.

5 Allgemeine Ergebnisse liegen zur Befragung aus dem ersten (Buschle und Meyer 2020) sowie dem zweiten Lockdown (Meyer und Alsago 2021a) vor. Außerdem existieren auch spezifische Sonderauswertungen zur gesamten Kinder- und Jugendhilfe im zweiten Lockdown (Meyer und Alsago 2021b), den Digitalisierungsprozessen in der Sozialen Arbeit während der Corona-Pandemie (Meyer und Alsago 2021c) sowie erste internationale Vergleiche (Schell-Kiehl et al. 2021).

6 Während in der ersten Befragung (Buschle und Meyer 2020) noch Fragen ausgelassen und deshalb unterschiedliche Stichprobengrößen (n) für die verschiedenen Antworten möglich sind, ist dies in der zweiten Befragungswelle (Meyer und Alsago 2021a) nicht mehr möglich gewesen. Allen Antworten liegen die gleichen Stichprobengrößen zugrunde.

7 Bei der Frage der Systemrelevanz von Mitarbeitenden des ASD zeigen sich in den Daten erhebliche Unterschiede zwischen den Bundesländern.

8 „Gleichzeitig sind die Beschäftigten in der Kinder- und Jugendhilfe sowie in der Sozialen Arbeit insgesamt durch die Arbeitsverdichtung extrem belastet: 66 % fühlen sich belastet oder extrem belastet und gut ein Viertel der Befragten (25,5 %) gibt an, keine Kraft mehr für die Weiterarbeit unter Corona-Bedingungen aufzubringen. Im Ergebnis denken 30,7 % über einen Stellenwechsel und 17,6 % sogar über einen Berufswechsel nach“ (Meyer und Alsago 2021b, S. 76).

9 Lediglich im Fall der Zusammenarbeit mit Vorgesetzten, Kolleg_innen sowie Kooperationspartner_innen besteht eine relative Gleichheit in der Einschätzung.
